# A unique case of urinary bladder simple melanosis: a case report and review of the literature

**DOI:** 10.1186/1746-1596-4-24

**Published:** 2009-07-22

**Authors:** Bo Jin, Syed Y Zaidi, Melvin Hollowell, Christopher Hollowell, Husain Saleh

**Affiliations:** 1 Department of Pathology, Sinai-Grace Hospital/Detroit Medical Center, Wayne State University School of Medicine, 6071 West Outer Drive, Detroit, MI 48235 USA; 2 Department of Urology, Sinai-Grace Hospital/Detroit Medical Center, Wayne State University School of Medicine, 6071 West Outer Drive, Detroit, MI 48235 USA

## Abstract

Melanosis refers to abnormal or excessive deposition of melanin pigment in the cells and/or tissue, which can be seen in any organ but commonly in skin and oral mucosa. Melanosis of the urinary bladder is an extremely rare benign condition and only a handful of cases been reported in the English literature before. In this article, we report a new case of urinary melanosis, describe the differential diagnostic features from pseudomelanosis and offer clues for correct diagnosis. We also provide comprehensive review of the literature on the subject.

## Background

Melanosis refers to abnormal/excessive deposits of melanin in the cells and/or tissue. This condition can be seen in any organ but more frequently in the skin and oral mucosa. Melanosis of the urinary bladder is a very rare benign condition and only 6 cases have been reported in English literature thus far [1-4]. Here, we report a new case, review the previously reported cases and discuss the criteria for making correct diagnosis.

## Case presentation

The patient is a 77 year-old African-American woman with medical history of urinary incontinence, large volume leakage, and severe urgency with leakage. She has been using diapers for the past two years. She was on Detrol LA for six months but no improvement of her symptoms. The patient was referred to our institution for further treatment. Under cystoscopy examination, clusters of punctuate dark spots were observed on the right lateral aspect of the bladder floor. There were no other lesions identified. Biopsies were obtained from the dark spots, and urine was sent for cytology. The patient had no previous cystoscopy examination or any other pigmented lesions on the skin or mucosal surfaces.

## Results

### Pathologic findings

**Figure 1:**
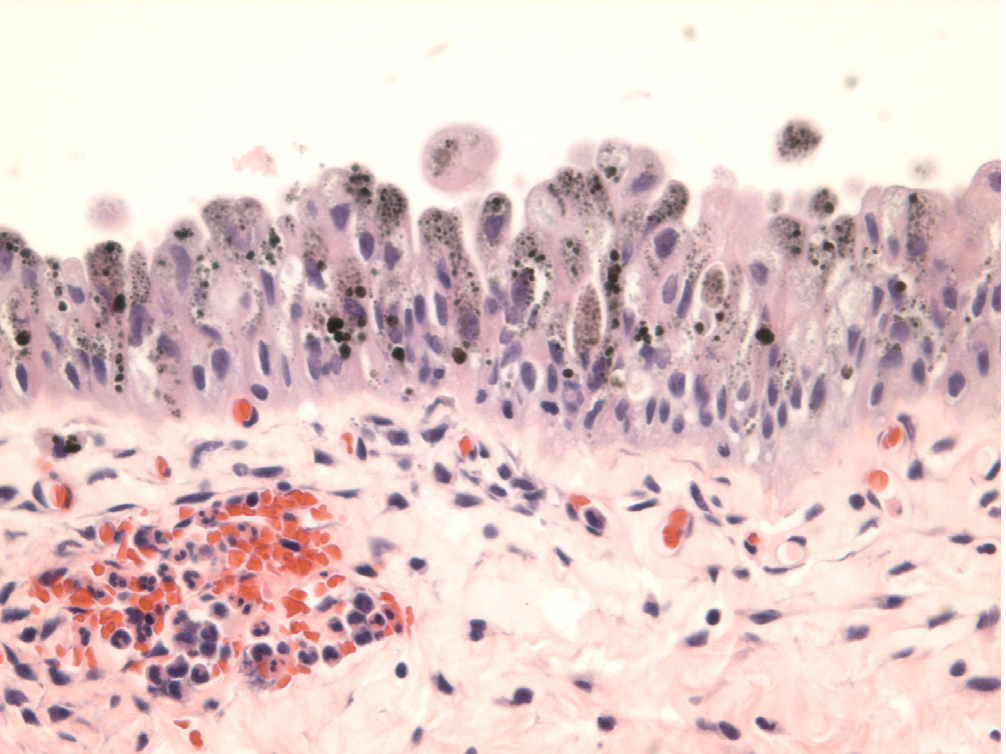
**Light brown powdery pigments to dark brown globules are mainly present in the cytoplasm, and partially or completely obscuring the nuclei**. There are no pigment deposits in the lamina propria.

**Figure 2:**
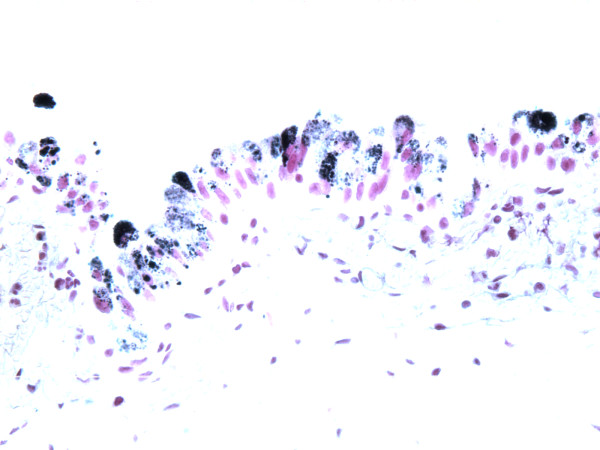
**The pigments stain black by Fontana Mason stain, consistent with melanin**.

The biopsy specimen consisted of multiple tan soft tissue bits ranging from 0.1 to 0.4 cm in greatest dimension. Microscopically, the sections showed benign urothelial mucosa with brown pigments focally present in the cytoplasm of urothelial cells. The pigments ranged from relatively light brown powdery to dark brown globules. The pigments were seen mainly in the cytoplasm and were partially or completely obscuring the nuclei. The underlying lamina propria showed mild congestion and acute inflammation with no pigment deposit (Figure 1). Special stains including Fontana Masson, Periodic Acid Schiff (PAS) and Gomori's iron stain were performed. The pigments were found to be positive by Fontana stain, consistent with melanin (Figure 2), while the stains for PAS and Gomori's iron were negative. The pigments disappeared after bleach procedure, further confirming the presence of melanin. There were no melanocytes identified in Hematoxylin & Eosin stained sections, or immunohistochemical stained sections for S100 protein, and HMB45 (human melanosome). The case was diagnosed as urinary melanosis. The urine cytology was negative for malignant cells and no pigmented urothelial cells were seen.

## Discussion

Urinary melanosis is a very rare lesion and the exact incidence can not be determined. We reviewed all the available previously reported urothelial melanosis in the English literature. All patients in reported cases have some associated urinary symptoms such as, hematuria, difficulty voiding, or urinary incontinence. The age of the patients ranges from 43 to 86 years old and both sexes have been affected.

Cystoscopically, brown to black pigmentation of the urinary bladder mucosa with flat or punctate patterns is usually observed. The pigmentation can be seen in any location of the bladder. There has been no description of associated mass lesions.

Histologically, dark brown pigments are present mainly in the urothelial cells, but sometimes few pigment deposits are also seen in the lamina propria. The pigments are non-refractile, powdery and can form variable sized globules. The pigments are positive by Fontana Masson stain, and negative for PAS and Gomori's or Lillie's iron stains. The melanin pigment disappears after melanin bleach treatment. In one case, melanocytes were seen admixed with urothelial cells appreciated by S100 protein immunohistochemical stain, but not in the H&E sections [2]. No nuclear atypia or melanoma was identified in any of the described patients (Additional file 1).

Melanin pigments should be differentiated from lipofuscin and hemosiderin deposits. Lipofuscin deposits are referred to as lipofuscinosis or pseudomelanosis in order to separate them from true melanosis (melanin deposits). Lipofuscin forms brown fine granules usually perinuclear located, representing undigested material from lipid peroxidation and associated with aging. It is positive by PAS stain, negative by Fontana Mason and iron stains, and does not disappear after bleaching procedure (bleaching resistant). Lipofuscinosis of the urinary bladder has been reported in a patient with interstitial cystitis after long term treatment with ciprofloxacin [5]. The urinary bladder showed scattered brownish discolorations under cystoscopic examination and the histological sections showed pigment granules in the urothelial cells and in the lamina propria histocytes. The pigment was PAS positive and bleach resistant. It was speculated that administration of ciprofloxacin might be the underlying cause of urinary lipofuscinosis due to free radicals generated by metabolism of ciprofloxacin. Lipofuscinosis of the urinary bladder have been also reported in patients with phenacetin abuse [6], which interestingly, also promote generation of free radicals during metabolism.

Comparing to lipofuscin deposit in the urinary bladder, lipofusion deposit is more commonly encountered in the colonic biopsy specimen as melanosis coli and are usually associated with anthracene containing laxative or herbal remedies usage. The colonic mucosa usually shows brown discoloration under colonoscopy examination, and histologically, it shows pigmented macrophages in the lamina propria. The term of melanosis coli is misleading, because the pigments are not melanin at all [7]. In this situation, the term pseudomelanosis coli or lipofuscinosis coli may be more appropriate to reflect the etiology of the colonic pigmentation.

Hemosiderin is an intracellular storage form of iron that is produced by phagocytic digestion of hematin. They appear as a golden yellow-brown intracellular or extracellular pigment under light microscopic examination, and pigments are positive by Gomori's or other iron stains. The excessive iron storage in tissue is known as hemosiderosis. Hemosiderosis of urinary bladder was reported in a man with multiple blood transfusions as part of treatment for his anaplastic anemia. Cystoscopic examination showed a brown black 2.0 cm polypoid lesion on the right lateral wall and surrounding numerous smaller lesions of similar appearance. Histologically, the urothelial mucosa showed prominent hemosiderin deposits and hemosiderin-laden macrophages in the lamina propria with unremarkable urothelium [8]. Although hemosiderin is mostly present in the stroma and macrophages, but not in the urothelial cells, it still should be included in the differential diagnosis for complete work up.

Most importantly, urinary melanosis needs to be differentiated from melanoma, which is also extremely rare in the urinary bladder, and classified as primary or metastatic, based on patient's clinical presentation and the presence or absence of atypical melanocytes in the urothelium [9]. In the absence of cutaneous or other visceral melanoma and the presence of atypical melanocytes in the urothelium adjacent to melanoma, the tumor is classified as primary. In a case report of primary melanoma of the urinary bladder, the patient had gross hematuria for four months and black mass lesions of the urinary bladder were seen under cystoscopic examination. The histology sections showed spindle and epithelioid heavily pigmented malignant melanocytes with high mitotic rate. Atypical melanocytes were also present in the urothelium [10]. The tumor cells were positive for S100 protein and HMB45. Therefore, being aware of clinical history, carefully reviewing histological features and performing necessary immunohistochemical stains, are very important in making the correct diagnosis. Urinary bladder melanosis is a benign condition of unknown clinical significance. One patient has been followed up for ten years and shows no development of malignancy [11].

Patients with melanosis, lipofucinosis, hemosiderosis, or even melanoma of the urinary bladder, can have similar non-specific symptoms such as hematuria, cystitis, difficulty voiding, or urinary incontinence. Under the cystoscopic examination, all show pigmented lesions, although melanoma may have associated mass. Histological examination is the most important way to accurately classify the pigmented lesions in the urinary bladder.

Melanosis of the skin or oral mucosa has been thoroughly studied because it is common. It can be seen in many conditions and is related to sun exposure (skin) or trauma (mucosa). It appears as gray or black macules. Histologically, it shows melanin deposits in the basal layer of the squamous epithelium. Melanosis of oral mucosa can also be seen in smokers and in patients with Peutz-Jeghers syndrome [12].

Unlike melanosis of the skin and oral mucosa, the underlying etiology of urinary melanosis is not yet established. Since no melanocytes are normally present in the urothelium or metaplastic epithelium, melanocyte ectopia has been speculated for the primary melanoma of urinary bladder [9], which may also apply to the development of urinary melanosis.

## Conclusion

Urinary melanosis is believed to be a benign lesion. Some author proposed melanosis vesica [1] for this condition and some just used the term of simple melanosis [2]. The latter may be more appropriate to reflect the benign nature of this condition. Careful review of histological features, applying special stains including Fontana Mason, iron, and PAS, bleach treatment, and immunohistochemical stains for S100 protein and HMB45 will lead to correct diagnosis. Treatment of the patient's underlying conditions and close follow-up with annual cystoscopy is recommended by some authors.

## Consent

Written informed consent was obtained from the patient for publication of this case report and any accompanying images. A copy of the written consent is available for review by the Editor-in-Chief of this journal.

## Competing interests

The authors declare that they have no competing interests.

## Authors' contributions

BJ participated in conception of the idea, writing of the manuscript and interpretation histological slides. SYZ participated in interpretation histological slides. MH and CH participated in clinical data collection. HS participated in conception of the idea and interpretation histological slides.

## Supplementary Material

Additional file 1 Summary of reported cases Click here for file
