# Supplementary material for: A unique case of urinary bladder simple melanosis: a case report and review of the literature
Source: Diagn Pathol. 2009 Jul 22;4:24. doi: 10.1186/1746-1596-4-24 (PMC2722577; doi:10.1186/1746-1596-4-24)
Supplement: Additional file 1 — Summary of reported cases [file 1746-1596-4-24-S1.doc]

| **Table 1**  **Summary of Reported Cases** | | | | | | | | |
| --- | --- | --- | --- | --- | --- | --- | --- | --- |
| # | Age/sex | Symptoms | Cystoscopy | Cytology | Histology | Special Stains | Melanin Bleach | S100/ HMB45 |
| 1 | 86-year-old white female | Urinary incontinence | Dark speckles involving bilateral bladder wall | Atypical transitional cells with intracytoplasmic brownish pigment | Urothelial intracytoplasmic brown pigment and melanophages in lamina propria | FMS Pos  PAS Neg  Iron Neg | Sensitive | Neg |
| 2 | 44-year-old white female | Recurrent cystitis, difficulty voiding asymptomatic gross hematuria | Black and white spotted mucosa | Not reported | Urothelial intracytoplasmic brown pigment | Lillie's reaction Pos | n/a | Pos |
| 3 | 43-year-old white male | Asymptomatic gross hematuria | Multiple flat irregular small foci of black pigmentation involving entire bladder | Not reported | Urothelial intracytoplasmic brown pigment | Lillie's reaction Pos | n/a | Pos |
| 4 | 72-year-old male | Dysuria, smelly urine | Black pigmentation dotted pattern involving entire bladder | Not reported | Urothelial intracytoplasmic brown pigment and brown pigment in lamina propria | Perl's iron Neg PAS Neg | Sensitive | Neg |
| 5 | 71-year-old white male | Asymptomatic bloody urethral discharge, mild physical exertion | Multiple flat irregular small foci of dark brown pigmentation | Not reported | Golden brown slightly refractile granules in the urothelial cells of bladder and urethra | FMS Pos Perl's iron Neg PAS Neg | Sensitive | n/a |
| 6 | 72-year-old white male | Urinary obstruction | >100 irregular flat foci of black mucosal pigmentation, involving entire bladder except trigone | Not reported | Golden brown slightly refractile granules in the urothelial cells of bladder | FMS Pos Perl's iron Neg PAS Neg | Sensitive | n/a |
| 7 | 77-year-old African-American female | Urinary frequency stress incontinence | Punctuate dark spots on bladder floor toward right lateral wall | No pigmented cells in pre and post cystoscopy urine cytology specimen | Urothelial intracytoplasmic light brown powdery to dark brown granules | FMS Pos PAS Neg Gomori's iron Neg | Sensitive | Neg |
| FMS: Fontana Masson stain, PAS: Periodic Acid Schiff stain, Pos: positive, Neg: negative, n/a: not applicable.  Correspondence between case number and references: 1-1, 2&3-2, 4-3, 5&6-4, 7-current case. | | | | | | | | |
